# Imprinting Technology in Electrochemical Biomimetic Sensors

**DOI:** 10.3390/s17030523

**Published:** 2017-03-06

**Authors:** Manuela F. Frasco, Liliana A. A. N. A. Truta, M. Goreti F. Sales, Felismina T. C. Moreira

**Affiliations:** BioMark-CINTESIS/ISEP, School of Engineering, Polytechnic Institute of Porto, 4200-072 Porto, Portugal; mtbff@isep.ipp.pt (M.F.F.); laant@isep.ipp.pt (L.A.A.N.A.T.); mgf@isep.ipp.pt (M.G.F.S.)

**Keywords:** molecularly imprinted polymers, electrochemistry, biomimetic sensors

## Abstract

Biosensors are a promising tool offering the possibility of low cost and fast analytical screening in point-of-care diagnostics and for on-site detection in the field. Most biosensors in routine use ensure their selectivity/specificity by including natural receptors as biorecognition element. These materials are however too expensive and hard to obtain for every biochemical molecule of interest in environmental and clinical practice. Molecularly imprinted polymers have emerged through time as an alternative to natural antibodies in biosensors. In theory, these materials are stable and robust, presenting much higher capacity to resist to harsher conditions of pH, temperature, pressure or organic solvents. In addition, these synthetic materials are much cheaper than their natural counterparts while offering equivalent affinity and sensitivity in the molecular recognition of the target analyte. Imprinting technology and biosensors have met quite recently, relying mostly on electrochemical detection and enabling a direct reading of different analytes, while promoting significant advances in various fields of use. Thus, this review encompasses such developments and describes a general overview for building promising biomimetic materials as biorecognition elements in electrochemical sensors. It includes different molecular imprinting strategies such as the choice of polymer material, imprinting methodology and assembly on the transduction platform. Their interface with the most recent nanostructured supports acting as standard conductive materials within electrochemical biomimetic sensors is pointed out.

## 1. Introduction

A biomimetic strategy enabling synthetic materials to mimic molecules ranging from amino acids and drugs to much larger proteins, bacteriophage or microbial cells, is the basis of molecular imprinting technology. The ability of imprinted materials to recognize the target analyte is comparable to natural molecular recognition in terms of affinity and sensitivity. Thus, designing biomimetic systems and synthetic analogues of biological processes are the focus of special attention in several research areas.

Molecularly imprinted polymers (MIPs) have the ability to recognize biological compounds like proteins [1,2,3,4], amino acids [5,6,7], peptides [8,9,10,11] or nucleotides [12], and chemicals such as pollutants [13], drugs and food additives [14,15,16]. Moreover, they can be applied in separation and purification processes [17], chemical sensors [18], catalysis [19] and drug delivery [20].

**Figure 1:**
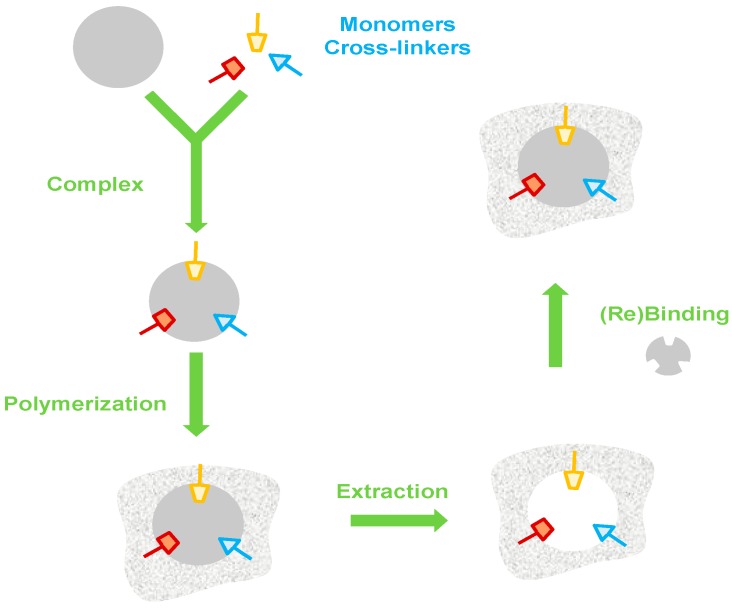
Synthesis of molecularly imprinted polymers.

Synthetic materials as biological analogue receptors have been highlighted in different research areas with great advances in sensor design [21]. MIP production is generally based on some form of template-directed synthesis. The template and functional monomers interact to form a complex during the imprinting polymerization and rebinding [22]. The polymerization starts with addition of the cross-linker and initiator. In the last step, the template is removed and the 3-D functional matrix keeps its geometry and organization guaranteeing the ability of MIPs to rebind the target molecule [23] (Figure 1).

Electrochemical sensors with MIP as recognition element combine outstanding characteristics. The biomimetic recognition secures their high selectivity, while the electrochemical transduction offers a highly sensitive response, i.e., the lowest possible limit of detection (LOD), in a short time [2,24]. The reusable electrochemical imprinted device produces a signal upon the selective interaction with the analyte. The electrical signal is proportional to the concentration of the target under analysis. Considering MIP construction, both the selection of polymeric materials and suitable imprinting processes for a target molecule are critical steps, especially considering the low detection levels required. Moreover, for combining MIPs with electrochemical sensors, different imprinting approaches (e.g., in situ bulk polymerization, epitope imprinting, surface imprinting, multi-template imprinting) can be employed [25,26,27,28].

The successful development of electrochemical sensors entailing the advantageous elimination of sample preparation requires multidisciplinary fields of analytical chemistry. The evaluation of several parameters is crucial to assess the performance of the sensor. These include the sensitivity by analysing the LOD and the linear and dynamic ranges, as well as the selectivity of response in the presence of interfering substances [29,30]. Additional characteristics like response time, reproducibility, stability, reusability and portability are also considered important key factors for evaluating any sensor.

It is possible to achieve higher sensitivity when developing a sensor by introducing some labels that bind to the recognition element or by applying indirect measurements. The main advantage for analyte detection is the signal amplification and the decrease of non-specific binding events [31]. This type of sensor is time consuming since it involves multiple steps, and it is also cost-limited. Thus, progress in label-free sensors able to directly detect the analyte allowing real-time measurements have been achieved [32,33]. However, these sensors lack amplification and need MIPs with very high affinity for the target as well as a very sensitive transducer. Very low LOD is in theory more challenging to achieve.

A general overview of the main polymeric materials and technological approaches for MIP design and fabrication are outlined first. In addition, this review describes the conjugation of MIPs as recognition element with transducer schemes, including immobilization strategies and labelling detection for different electrochemical sensors, providing an outline of electrochemically–based imprinted biomimetic sensors.

## 2. Polymeric Materials

Polymeric materials holding complimentary features in dimensions and functionality to the template are obtained by different routes. The type of interactions that occur during the phase of imprinting within the matrix-forming material, such as covalent bonds, non-covalent binding or metal-ion mediated imprinting can be customized according to the desired MIP, target bioanalytes and type of sensor, and has been extensively reviewed elsewhere [34,35,36]. Functional monomers are used to mediate specific chemical recognition processes. In addition, the monomers are held in place by cross-linking agents. The selective artificial recognition cavities are then formed within matrices or impressed on surfaces in appropriate solvents [37,38,39]. The type of interaction between the template and monomers determines the template removal method, either by a simple extraction or by chemical cleavage. Thus, choosing the starting material or composite with proper recognition ability can deliver molecular cavities complementary to the template in shape, size and function, accomplishing the desired selectivity [4,40].

### 2.1. Organic Polymers

Most formulations used in molecular imprinting technique for sensing purposes are based on the traditional free radical polymerization, relying on non-covalent interactions, such as electrostatic interactions or hydrogen bonding, between the template and functional groups of the organic monomers [41]. These interactions occur first in the pre-polymerization complex and the polymer is grown afterwards in the presence of a proper cross-linker and solvent type. It is a widespread method because the template has multiple potential binding sites to be targeted, together with the less chemistry involved when compared to the pre-synthesis of covalent adducts [34]. The mild reaction conditions of free radical polymerizations can be implemented using a myriad of vinyl monomers, including ethylene, styrene, methacrylic acid, acrylamide, among many others [34,42]. Methacrylic acid as functional monomer has found broad applicability, mainly because it is a carboxylic acid-based monomer and this group can serve as both a hydrogen bond donor and acceptor, and additionally it can establish ion-pair and dipole-dipole interactions [34]. The design of MIP synthesis should consider a number of variables taking into account the nature and levels of the various components of the mixtures, i.e., template, functional monomer, cross-linker, solvent and initiator, as well as the method of initiation, polymerization time and temperature [42]. A screening of functional vinyl monomers enables the copolymerization of two or more monomers with different functionalities, which is highly desirable to establish multiple types of interactions with the template. MIP synthesis using only non-covalent assembly in polar solvents, namely water, may face some difficulties because the electrostatic and hydrogen bonding interactions are weakened in the polar medium [41,43]. Thus, to achieve good affinity and selectivity, especially for imprinting biological molecules in aqueous medium, adjusting the polarity of the solvent with the template solubility is crucial [40]. Nevertheless, constructing MIPs with improved performance in organic polar solvents or water can be achieved by combining stronger interactions in such media, like hydrophobic and metal-coordination interactions [41,44].

### 2.2. Hydrogels

Hydrogel substrates are swellable soft materials of considerable interest. Nonetheless, producing chemically and mechanically stable hydrogel MIPs with high specific recognition is challenging [45]. This is due to the lower binding strength of the non-covalent interactions established between the template and monomers, crucial to obtain the imprinting outcome, in aqueous solutions. Among the acrylate family of polymers, polyacrylamide has been successfully used as an imprinting matrix for biological molecules. Along with water solubility, low cost, easy production and engineering are some of polyacrylamide gels’ attractive properties concerning molecular imprinting [46]. Studies on polyacrylamide hydrogel-based MIPs have demonstrated sensitivity and selectivity towards several proteins with different biological roles, sizes and electrochemical activities, namely haemoglobin, myoglobin, cytochrome c, bovine serum albumin (BSA) and catalase [47,48,49]. Further improvements in terms of MIP selectivity have been attempted by including a metal chelating complex comprising bifunctional vinyl groups with the ability to co-polymerize within the polyacrylamide matrix [50]. These advanced imprinted cavities with metal-coding for enhanced selective protein recognition showed higher BSA binding and selectivity. These results may be explained by a better macroporosity and stability of the polymer backbone, as well as additional metal contribution to favorable interactions with the protein [50]. Hydrogel polymers undergo a reversible volume transition between swollen and collapsed phases. Thus, they can be designed to respond with a phase transition as triggered by certain external stimuli, such as temperature, pH, and light, among others. These features of stimuli-responsive hydrogels could benefit MIPs performance by reducing non-specific binding and improving transport of the template [51]. The synthesis of smart hydrogel MIPs that can modulate their affinity for the target macromolecules enabling a switchable capacity for the binding and releasing processes according to external stimuli has attracted considerable research interest and has been comprehensively reviewed [52,53].

### 2.3. Sol-Gel Materials

The most highlighted properties of sol-gel processing when constructing matrices to detect an analyte with high sensitivity and selectivity are the ability to control the porosity and the nanostructuring while achieving the desired purity and homogeneity [54]. Systems with tailored features were first described using silica gels and since then, growing interest enabled expanding the use of these inorganic materials in MIP design, including hybrid organic-inorganic matrices, for various analytes [55,56,57,58]. Likewise, inorganic matrices of titanium oxide sol-gels have enabled the production of imprinted films with specific binding cavities with a variety of functionalities [59,60]. One of the advantages presented by titanium over silane sol-gels is the improved stability when exposed to higher temperatures [54]. Generally, the sol-gel process occurring at mild conditions consists of a system transition from liquid “sol” into solid “gel”. Alkoxide precursors are hydrolyzed and react with each other (or with the unhydrolyzed molecules), followed by polycondensation reactions that release water or alcohol when the matrix is cross-linking [54]. As the condensation propagates, there is a gradual increase of the matrix viscosity until originating an interconnected, homogeneous and rigid material. The degree of cross-linking and the network formation can be tuned because there are several parameters that affect the sol-gel reactions. An optimization requires considering the type of solvent and the pH of the solution, the catalyst chosen and its concentration, the reaction temperature and time, and the nature of the (R) group of the alkoxides [61,62]. Thus, when adjusting these variables, fundamental properties (e.g., refractive index, surface area, porosity, and mechanical features) can be established according to the goal of the sol-gel sensor [54]. For example, a silica sol-gel thin film molecularly imprinted was used to coat a piezoelectric sensor, and its ability to determine the presence of amino acids, namely l-histidine, has been described. The imprinted polymer obtained without pre-protection of the amino acid exhibited excellent properties in terms of stereoselectivity, specificity and stability [55]. The flexibility and low density properties are advantageous since the thin film is fabricated using mild polymerization conditions without decomposition problems and guarantying excellent selectivity [55]. Other studies include the optimization of sol-gel based MIPs to recognize molecules like metabolites, proteins, cells, pesticides [61,63,64,65].

## 3. Imprinting Technology

MIPs denote polymer matrices designed for highly selective recognition of target molecules. The ideal sensitivity for a certain envisioned application can be achieved with a proper control of the polymer matrix and assuring an optimal design of the imprinting process. An important role is played by the conditions under which the template molecules are introduced to the polymerization mixture, i.e., appropriate procedures for imprinting the affinity sites with memory for the target are critical.

### 3.1. Bulk Imprinting

In the most usual imprinting approach, the surface of the transducer is coated with a pre-polymer mixture to which typically a small molecule template is added. These components interact in the solution creating a network that is then polymerized. The template that is entrapped within the polymer matrix in the curing process is removed by elution, leaving behind cavities throughout the bulk material that possess shape and size selectivity to recognize the analyte in the subsequent sensor measurements [3]. The binding sites in the imprinted material should retain their shape, thus the polymer needs to meet certain requirements in terms of porosity, allowing molecular diffusion, and also stiffness, obtained via suitable cross-linking [66]. To assist the process of template removal, physical (e.g., increasing the temperature) or (bio)chemical (e.g., washing with solvents, acids/bases, detergents, and digesting enzymes) methods are usually applied [67]. Due to the creation of binding sites within the polymeric bulk, the features of this type of imprinting are most convenient to generate binding cavities to relatively small molecules. The advantages for macromolecules would be related to the formation of 3-D binding sites for the entire structure with easy preparation procedures. However, the drawbacks are linked to the bulk volume, originating poor mass transfer and slow binding kinetics due to long diffusion path lengths and reduced number of effective imprinted sites in the polymer matrices for template rebinding [3,68]. These limitations are reflected in the type of molecules that are imprinted, usually restricted to small molecules [69]. Thus, alternative methods are proposed to place the binding sites mainly on the surface, and thus enlarging the applications with enhanced efficiency.

### 3.2. Surface Imprinting

In order to extend the use of MIPs to larger molecules, allowing their diffusion and facilitating template removal and rebinding, alternatives have been conceived, such as: (a) post-processing of bulk imprinted material by grinding and sieving to expose the imprinted sites; (b) imprinting hydrogels; (c) adding substances to increase the porosity of the polymer; (d) increase the surface-to-volume ratio by using polymer nanoparticles; (e) use a conducting polymer film deposited by electropolymerization [67,68]. In these approaches, the binding sites that are imprinted are positioned near the polymer surface and allow the synthesis of thin polymer films. Due to the existence of the support, the imprinted assembly is more robust and is easily integrated within electrochemical platforms. The main drawback is related to the available surface that is limiting the extent of binding sites. Nonetheless, these materials offer expedite accessibility to larger target analytes, namely proteins, virus and cells. Different approaches towards the production of surface imprinted materials have been described in the literature and presented next.

#### 3.2.1. Microcontact Imprinting

**Figure 2:**
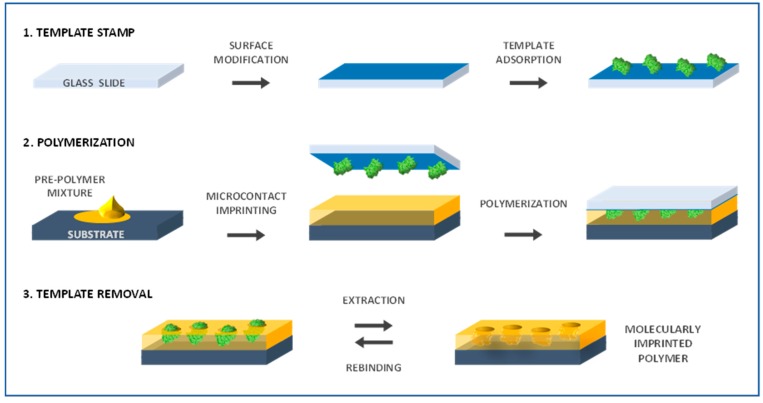
Schematic representation of microcontact imprinted polymer fabrication.

Included in the soft lithography techniques, microcontact printing is commonly used to transfer molecules onto a surface using a patterned elastomeric mold or stamp (e.g., poly(dimethylsiloxane)—PDMS). This simple and cost-effective technique is very attractive taking also into account the versatility of substrates and materials to be imprinted [70]. Briefly, the stamp, previously molded from a solid master, is coated with biomolecule-containing solution and then brought into contact with the substrate to be patterned; in this stage, it transfers the biomolecules from the microscale raised surfaces in the mold to the substrate [71]. As previously mentioned, the creation of artificial recognition sites by molecular imprinting is comparable to natural receptors with the additional advantage of resisting much harsher conditions. To achieve successful specific molecular recognition, factors like size, shape and chemical functionality have to be addressed. Thus, when considering macromolecules, such as proteins and cells, the use of bulk monoliths to form the imprinted sites may hinder a proper recognition due to diffusion constraints [3,72]. In this context, microcontact printing techniques can be useful to form highly sensitive, low cost and stable thin films with imprinted surface molecular recognition architectures [73]. Biomimetic sensors incorporating microcontact imprinted films for various protein biomarkers (e.g., C-reactive protein, ribonuclease, lysozyme, myoglobin, ovalbumin) have been studied [73,74]. The general procedure of extended standard microcontact printing technique applied to MIP construction can be depicted as (Figure 2): a glass slide is used to make the template stamp by its adsorption on pre-treated glass surface, while the pre-polymer (monomers and cross-linker) mixture solution is deposited in the transducer support serving as MIP substrate; the template stamp is brought into contact with the substrate and the polymerization is initiated; the last step involves removing the stamp and eluting the bound template from the obtained MIP [75].

The flat and optically transparent glass used as the adsorption substrate enables the sensing layer to be synthesized by UV polymerization. Also, the glass surface is easily modified with organic compounds (e.g., amino groups) to enhance its affinity for the template biomolecules. In order to increase the site-specific spatial organization of the functional monomer, the template can be first assembled with the monomer in the cover glass slip and only after introduced into the remaining polymer mixture with subsequent polymerization. Once the analyte molecules interact with the sensor surface they can easily rebind to the recognition cavities. This type of procedure has been used to create high affinity MIPs for several biomolecules, such as ribonuclease A (RNase A), lysozyme and myoglobin [75,76]. One study used microcontact imprinting to prepare capacitive gold electrodes for BSA detection as a model protein. BSA was imprinted onto pre-modified gold electrode surface by microcontact with subsequent UV-polymerization and the results showed good selectivity and stability [77]. As for other types of imprinting techniques, the functional monomer(s) chosen, which mimic structures or chemical features of natural receptors, the cross-linking agent and the respective ratios are very important in achieving higher selectivity for the target molecule [73,78]. Moreover, in terms of MIP recognition of protein targets, evidence suggests that conformational changes may occur, along with protein denaturation leading to some degree of aggregation, thus recognition of protein fragments may take place to some extent [76]. An example of such occurrence was described for RNase A when interacting with the functional monomer styrene, that despite providing MIPs with higher affinity for the enzyme could lead to RNase A denaturation. The study of surface morphology and imprinted cavities suggests the presence of large sizes of imprinted cavities fitting with the hypothesis that aggregates rather than individual RNase molecules bind into MIPs cavities [76]. Nonetheless, good recognition is not of concern and the presence of any small structural fragments can work also as recognition elements [75]. Microcontact printing has also been combined with layer-by-layer (LbL) assembly to have an unconventional approach towards the concept of molecular imprinting, simultaneously tackling the current difficulties of slow binding kinetics and low accessibility of binding sites in the bulk polymer [79]. This method has been advanced to introduce new functionalities to nanostructured LbL thin films, namely to fabricate surface molecularly imprinted films [79]. Interesting results have also been reported on the use of this methodology to prepare bacterial stamps and proceed to the imprinting and quantification of bacterial cells [80,81].

#### 3.2.2. Polymer-Brush Imprinting

**Figure 3:**
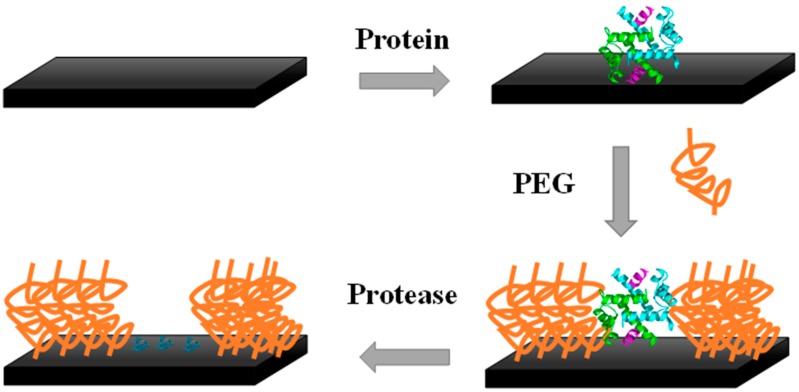
Polymer-brush imprinting.

Polymer-brush imprinting relies on the tethering (grafting) to a solid interface of polymer chains through one end. The template molecules are first attached to a polymer layer with subsequent removal by enzymatic/chemical treatment. A grafted polymer occupies the space neighboring the adsorbed template, and when the target analyte recognizes and rebinds to the substrate, it only occurs in the imprinted cavities [82] (Figure 3).

In the particular case of electrochemical biosensors, the main challenge is to tackle the tendency of abundant proteins present in the complex mixture of compounds that compose biological samples to physically adsorb without specific receptor-recognition interactions. This adsorption is non-specific and it naturally reduces the expected biosensor performance leading to electrode fouling and decreasing the sensitivity when it comes to detect the target biomarkers typically in a very low concentration. Non-specific interactions can be prevented or minimized by using self-assembled monolayers (SAMs), namely of poly(ethylene glycol) (PEG) or oligo(ethylene glycol) (OEG) [82,83,84,85].

Zdyrko et al. [86] employed grafting of polymer brushes as an approach to surface protein imprinting. An ultrathin polymer layer of poly(glycidyl methacrylate) served to chemically bind the protein molecules that were later removed by protease treatment. A PEG grafted layer was introduced on the surrounding space of the adsorbed molecules, so that the residual amino acids on the surface after template removal formed nanosized recognition sites. The imprinted substrate was selective, differentiating the bovine serum fibrinogen from BSA [86]. Another method of surface imprinting using SAMs was showed by Wang et al. [87] by producing a thiol film on a gold surface with the co-adsorption of the template biomolecules. The template is removed afterwards, leaving the SAMs matrix with recognition sites. This system was used to detect the carcinoembryonic antigen, a cancer biomarker, in samples containing only the purified biomarker and also using the culture medium of a human colon cancer cell line. More recently, Yang and co-authors used silica particles to anchor proteins and polymer brushes through a reaction of thiol—disulfide exchange. The grafted polymer brushes were temperature-sensitive and in their collapsed state above the lower critical solution temperature the protein molecules were protected what enabled to control their catalytic activity [88].

A device has been reported to detect uric acid (UA) using differential pulse cathodic stripping voltammetric (DPCSV) analysis [89]. The electrodes were prepared of sol-gel modified graphite and worked as support to graft MIP brushes of poly(melamine-co-chloranil). This approach enabled to avoid non-specific binding arising from cross-reactivity and matrix interactions. The DPCSV signals were generated by the electrochemical oxidation of UA with subsequent cathodic stripping, allowing a selective detection within the limits required to diagnose hyperurecemia. In a similar work developed to detect dopamine, Prasad et al. applied DPCSV in combination with a solid-phase microextraction fiber. In this method, MIP brushes prepared using SAMs of low molecular weight were grafted to a sol–gel matrix coupled to an optical fiber [90]. In summary, polymer brushes hold multi-faceted properties, like self-assembly, wettability, switchability, and biocompatibility.

#### 3.2.3. Surface Grafting

Surface grafting imprinting is another effective approach to overcome the difficulty of template mass transfer within MIPs and particularly valuable when considering the imprinting of macromolecular structures such as proteins, polysaccharides or microorganisms [91].

Biomimetic sensor development can highly benefit from surface protein-imprinted nanoparticles. The imprinted material can be obtained through the synthesis of a thin polymer film or by using a substrate (e.g., nanoparticles, nanotubes) that functions as support to attach the template to its surface with following polymerization around it [1,2,92,93]. The imprinted binding sites are positioned nearby the surface of the outer polymer layer, avoiding limited mass transfer and enabling easy template removal [93,94]. The drawback is the limited number of binding sites.

Kryscio and Peppas described several macromolecular imprinting works published through 1994–2010, showing a considerable rise of interest since 2005. This critical review shows that this particularly field has been ruled by surface imprinting that comprises around 60% of the overall published works [3]. Surface imprinting techniques are widely applicable to proteins that have great interest as disease biomarkers. In this context, surface grafting MIPs present some advantageous properties because the presence of a support confers a higher physical robustness and it is more easily integrated in sensory platforms. Still, as the protein is only partially imprinted, some loss in specificity can be a drawback. Some studies using this approach in combination with electrochemical transduction are highlighted below.

Moreira et al. [1] described a MIP on the surface of multiwalled carbon nanotubes (MWCNT-COOH) that functioned as an artificial antibody for the selective detection of troponin T. The protein was covalently linked to MWCNT surface and a polymer prepared with acrylamide and *N*,*N*′-methylenebisacrylamide filled the vacant spaces. The protein was removed from the polymeric matrix by acidic treatment with oxalic acid. The electroactive modified nanotubes were incorporated in a PVC/plasticizer mixture forming a membrane, and the performance of the biomimetic sensor was subsequently tested. In general, these simply designed sensors offer excellent properties in terms of short measurement times, high precision, accuracy and throughput, as well as low LOD, and good selectivity [1].

**Figure 4:**
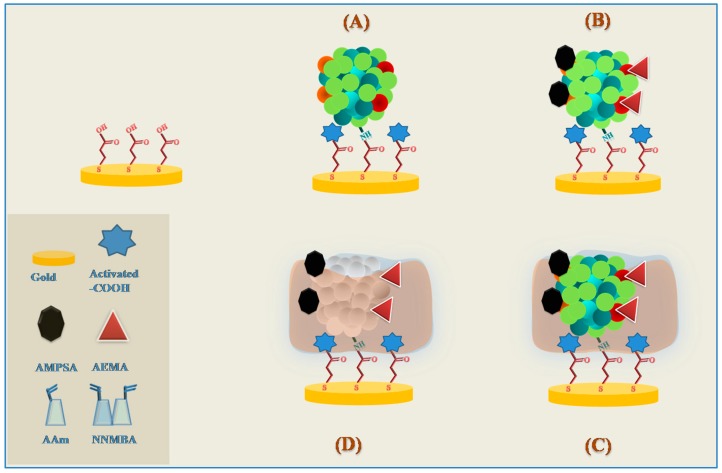
Synthesis of the SPAM material. (**A**) Protein bound; (**B**) Charged labels; (**C**) Polymerization; (**D**) Template removal.

The same authors reported more recently the synthesis of a smart plastic antibody material (SPAM) to target myoglobin in point-of-care. The design followed a bottom-up approach and the SPAM was tailored on top of disposable gold-screen printed electrodes (Au-SPE). The MIP cavities were visualized for the first time by AFM only in the SPAM network. Another outstanding feature was related to the rebinding of the target protein that only occurred in the SPAM materials, producing a linear electrical response against square wave voltammetry (SWV) assays. In contrast, the non-imprinted polymers showed a similar-to-random behaviour. The SPAM/Au-SPE device allowed the detection of myoglobin down to 1.5 µg/mL and 0.28 µg/mL in EIS and SWV, respectively. Using voltammetric assays, the SPAM materials showed negligible interference from troponin T, BSA and urea, displaying promising results for point-of-care applications [2] (Figure 4).

#### 3.2.4. Electropolymerization

Electropolymerization is typically conducted by applying a suitable potential or range of potentials to a solution containing the template with the monomer, originating a film formation on the surface of the electrode [95]. This simple approach is useful since by adjusting the electrochemical conditions (e.g., potential range, number of cycles and scan rate) and by using different conductive materials of various shape/size one can achieve a close control of the polymer thickness [18]. The electropolymerization around the template normally requires the use of a functional monomer, a porogenic solvent, and sometimes a cross-linking monomer in contact with the transducer surface. Also, no initiator is required, nor UV light or heat. Thus, electropolymerization is another method with plenty attractive features to overcome the difficulties of producing MIPs for macromolecules such as proteins, as reviewed elsewhere [96].

**Figure 5:**
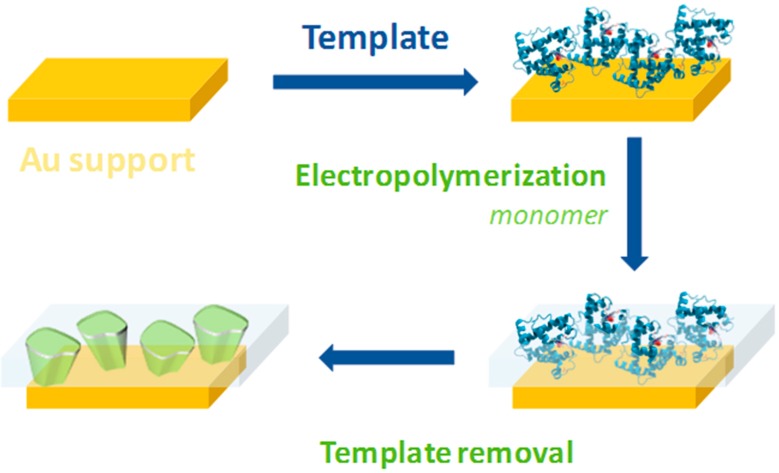
Electrosynthesis of MIPs.

The electrosynthesis of MIPs involves conductive polymers (ECP) and insulators/non-conductive polymers (NCP). The charge transfer between the electrode substrate and the analyte that occupies the molecular cavities of the MIP is assured by incorporating ECP matrices in the film. Several research works in the literature report the use of electroactive monomers like 3,4-ethylenedioxythiophene (EDOT) [97,98,99], pyrrole [100,101,102,103], aniline [104,105], thiophene [106,107], and dopamine [108,109] for MIP electrosynthesis of organic compounds (Figure 5).

Several non-conducting MIP films from the electro-synthesis of non-conductive monomers as phenol [32], aminophenol [110,111], phenylenediamine [112,113] among others have also been reported. Electropolymerization resulting in non-conducting MIP films are widely used for capacity chemosensors specially polyphenylenediamine and polyphenol. These materials form non-conductive, compact MIP films after electropolymerization. The prepared MIP biorecognition element can detect the analyte at nanoscale level with notable selectivity and low LOD. The selectivity of the electrosynthesized MIP can be improved by modifying the monomers with additional functional groups.

The resulting conducting or non-conducting MIPs show advantages and disadvantages. The deposition by electropolymerization of non-conducting MIP films has to be tightly controlled related to the polymer thickness. This self-limiting characteristic is due to the need of stopping the deposition upon reaching a thickness at which the polymer insulates the fundamental conducting electrode surface. On the other hand, deposition of ECPs by electropolymerization may occur indeterminately as the deposition conditions control the polymer thickness. The method of choice for signal transduction is related with the conductivity of the polymer. The impedance variations can more easily be detected with a NCP.

### 3.3. Epitope Imprinting

Another approach for macromolecular imprinting that relies on using a partial, short peptide as template, has been designed namely to improve the recognition of proteins and polypeptides. In the epitope strategy, that closely mimics the natural recognition antigen-antibody, suitable moieties have to be properly identified to represent the parent macromolecule [114]. Short fragments from exposed domains of the proteins have been generally selected as template. The use of epitope imprinting can thus be particularly valuable to target membrane proteins, since the recognition occurs through the part of the protein localized on the cell surface [115]. Interestingly, this approach is very sensitive to any mismatch in the amino acid sequence, as determined by imprinting native and single-point mutated template peptides for BSA. This experience demonstrated that the MIP film imprinted with the mismatched peptides presented lower selectivity of detection [116]. Similar results were obtained when imprinting target peptides relevant for cancer diagnosis, since the MIP was able to differentiate single amino acid mismatches [117].

Some advantages are attributed to this process, namely because it is a small template that is impressed, reducing non-specific interactions pertain to biomolecules complexity as well as conformational instability and improved binding kinetics [3,116,118]. Nonetheless, as MIPs are able to bind the whole analyte, diffusion constraints on rebinding studies have to be addressed and a combination with surface imprinting techniques facilitates protein access and defined orientation [117,119]. Such epitope-based imprinted materials show stronger affinity to the target protein with prominent selectivity when performing competitive binding assays [118,119]. Detection of cytochrome c is an example of the successful use of epitope imprinting together with electrochemical template removal and control of polymer film thickness [120]. Several studies show that the epitope approach combined with surface-confined imprinting using hybrid nanostructured materials can provide improved protein recognition [121,122].

## 4. Electrochemical Transduction

In biosensors with electrochemical transduction the target analyte binds the biorecognition element selectively. The detection of the analyte is based in the variations at electrode surface in terms of alteration in current and or voltage.

The first MIP-based electrochemical sensor was described in the early 1990s by Mosbach’s group [123]. Three years later, Hedborg and co-authors reported the first thin polymer membrane-based molecular imprint. The membrane was composed by the imprinted sites of the l-phenylalanine anilide and was casted as a sensing material film in field-effect capacitors [124].

Electrochemical biosensors are widely synthesized by electropolymerization of such monomers as pyrrole [101], aniline [104], *o*-phenylenediamine (o-PD) [125,126], phenol [32], aminophenol [110,111,127] and EDOT [128,129], self-assembled monolayers (SAMs) [2,130,131] and sol-gel [93] materials.

**Figure 6:**
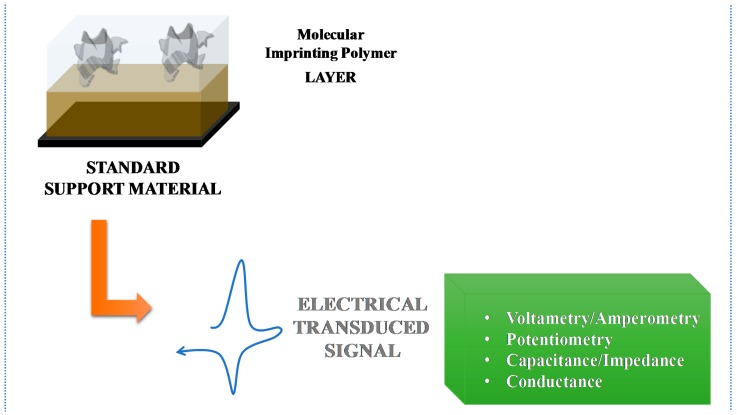
Basic mechanisms of electrochemical MIP-based biomimetic sensors.

The electrochemical transduction can be categorized according to the analytical signal output. In potentiometry the measurement is the potential (V), in amperometry and voltammetry is the current (A), in impedance is the resistance (Ohm) and in conductance, Siemens (S). Figure 6 illustrates the basic mechanisms of electrochemical MIP-based biomimetic sensors.

### 4.1. Voltammetry/Amperometry

In voltammetric/amperometric MIP-based sensors the electroactive species are detected directly involving a linear or logarithmic correlation between the amount of species and the current measured [18,132]. Voltammetry is widely used due to inherent oxidation and reduction potential features of the electrodes, sensory materials and target analytes. The voltammetric techniques usually applied include: cyclic voltammetry (CV) linear sweep voltammetry (LSV), differential pulse voltammetry (DPV), square wave voltammetry (SWV) and cronoamperometry (CA). Table 1 depicts a summary of various voltammetric/amperometric MIPs and their analytical features for different organic compounds.

Some researchers incorporated the redox probe in the polymeric matrix. One example is the work developed by Udomsap et al. [133] that reported a versatile electrochemical MIP-based sensor with the introduction of a redox probe (vinylferrocene) inside the binding cavities of a crosslinked MIP. The biomimetic sensor detected easily the analyte benzo[a]pyrene with LOD of 0.09 μmol/L [133].

Some efforts have been developed by several research groups by using different nanomaterials and polymers in order to obtain a stable, sensitive and selective electrochemical MIP sensor. The high surface to volume ratio presented by these materials is a property that helps the molecule to access the multiplicity of the binding sites.

In order to increase the sensitivity of the biomimetic materials, some metallic nanoparticles or nanomaterials can be added to the polymeric matrix. These include iron oxide, gold nanoparticles (AuNPs), silver, and graphene oxide, among others. An innovative biomimetic material fabricated with magnetic graphene oxide and AuNPs was created and applied as bioreceptor to build a dibutyl phthalate electrochemical device. Under optimized conditions, the sensor showed a LOD of 8.0 × 10^−10^ mol/L [134].

Recently, Tan and co-authors described an electrochemical MIP-based sensor synthesized on glassy carbon (GC) electrode surface for carbofuran detection. Graphene oxide and AuNPs composites were used to improve the analytical signal upon binding of the target analyte on MIP receptor. The polymerization around the template molecule was carried out with the presence of functional monomer (methyl acrylic acid) and cross-linker (ethylene glycol maleic rosinate acrylate). The LOD was 2.0 × 10^−8^ mol/L [135].

Moreover, magnetic nanoparticles have been incorporated in conductive polymer and the combination of both properties resulted in an enhancement of the sensitivity of the biomimetic sensor. This device was described by Zamora-Gálvez and co-authors for sulfonamide detection with a LOD of 1 × 10^−12^ mol/L with similar results to the obtained by liquid chromatography and mass spectrometry [92]. Another technique to improve the sensitivity of MIP sensors is the polymerization of the electroactive materials concomitantly with substrate-guided dopant immobilization [136].

### 4.2. Potentiometry

In potentiometry the potential is evaluated in flow or batch conditions. The potential obtained is used to evaluate or quantify the amount of an ion in solution [153]. Potentiometry shows some advantages in terms of simplicity, fast response time and low cost for detection of different compounds. Potentiometric sensors can be allocated in two groups: ion selective electrodes (ISEs) and field-effect transistors (FETs) [154]. In general, MIP potentiometric based sensors show outstanding features in terms of design, reusability, stability, low cost, response time and good sensitivity and selectivity.

#### 4.2.1. ISE Systems

In potentiometry the species do not need to diffuse through the membrane, thus there is no size constrain on the template molecule. Due to these benefits, several MIP-based sensors have been described with potentiometric transduction [155] for detection of organic and inorganic compounds (Table 2). Murray et al. stated for the first time an ion-selective electrode based on a MIP as a biorecognition element with potentiometric transduction [156] for lead ions selective detection.

The main concern of the ISE includes the use of a PVC membrane, as it needs high volatile solvents and the membrane thickness is difficult to control, it may cause a reproducibility problem at the time of production.

Some imprinted PVC membranes have been recently reported for some drugs and metabolites as: azithromycin [157], losartan [158], clenbuterol [159], taurine [160] and carnitine [162,169], among others. In these, a sensitive MIP for azithromycin has been reported. The polymerization occurred in the presence of the monomers, cross-linker, initiator and target analyte, being 2-vinyl pyridine and acrylic acid, ethylene glycol dimethacrylate, benzoyl peroxide and azithromycin respectively. After the polymerization, the sensor was entrapped in a polymeric membrane with di-butyl phosphate (DBP) and di-octyl phthalate (DOP) as solvent plasticizers. The sensor shows good overall analytical performance in terms of slope and LOD (49.1 and 51.3 mV/decade and 10 × 10^−7^ mol/L) respectively.

Imprinting proteins have also been successful integrated with potentiometric transduction. However, imprinting proteins can be a challenging task, since they change their conformation easily. Also, proteins hold multiple charge locations that change according to the specific conformation. In order to overcome these limitations Rebelo and co-authors proposed recently an imprinted sensor for prostate specific antigen detection, were the imprinted sites were obtained by adding charged monomers as labels around the template protein in order to obtain specific binding sites. The polymerization was carried out with uncharged monomers in neutral conditions. The sensors were entrapped in PVC membranes with a solvent plasticizer and applied to conventional solid-contact carbon electrodes. The analytical performance of the sensor was evaluated by potentiometric assays by means of calibration curve, selectivity study and sample analysis [167].

#### 4.2.2. FET Systems

The integration of MIP-based sensors with organic/inorganic compounds within an ion-selective field-effect transistor (ISFET) configuration is also a very attractive approach. The first report of ISFET was a short communication by Bergveld in 1970. This work described for the first time a miniaturized silicon-based chemical sensor, followed by a more extensive paper in 1972 [170,171]. FET biosensors provide huge advantages in terms of response time and dimension, enabling biosensor integration with on-chip arrays as a promising tool concerning the production of cheap and portable microanalysis devices. A work published in 2016 described the integration of MIP with ISFET for creatinine and urea. This biosensing device showed good reproducibility, repeatability and stability, as well as high selectivity [165]. Despite ISFET advantages, the classical FET systems may present compatibility limitations related to necessary chemical modifications when incorporating a recognition layer for sensing applications [172].

With the progress of research in technological applications, extended-gate field-effect transistor (EG-FET) has emerged, a practical and useful alternative as extension of ISFET that allows producing different sensors, including those based on MIP techniques. These devices based on the measurement of the current changes at constant gate voltage, allow the flexibility of shape of the extended gate structure, high sensitivity to distinguish homologous analytes, and good insensitivity to the light [172,173].

Several works have described EG-FET systems based on MIPs. In 2016, Dabrowski and co-authors reported a MIP film developed for d-arabitol detection in biological samples like urine using the EG-FET as transducer. The analyses were performed under stagnant-solution binding conditions, presenting a linear dynamic concentration range between 0.12 and 1.00 mmol/L with a LOD of 0.12 mmol/L. In general, these devices were considered good sensors able to detect very low concentrations of d-arabitol in urine, using a simple and inexpensive procedure [172]. Likewise, another work published by Iskierko and co-authors described two EG-FET based MIP sensors for a selective detection of specific phenylalanine enantiomers, d- and l-phenylalanine (Phe). Thus, the authors present successful sensors able to recognize levels of d- and l-Phe under 13 to 100 µmol/L with a LOD of 13 µmol/L and a good discrimination of interfering substances, such as d-proline, d-alanine, and d-tyrosine [174].

### 4.3. Capacitance/Impedance

The progress observed in electrochemical biosensors brought some promising research in capacitive and impedimetric approaches [175]. An impedimetric biosensor is defined as the application of impedance, as the transduction principle, in a biosensor system, which through measurements and/or monitoring of targeted analyte (antigen-antibody interactions, oligonucleotide-DNA interactions, among other biomolecules) on electrode surface, provides the output of an electrical impedance signal proportional to analyte activity [176].

Electrochemical Impedance Spectroscopy (EIS) allows monitoring of the electrochemical events occurring at the interface electrode and solution. This method allows the calculation of several parameters of the fitted model based on the ratio between the voltage and the current at a specific frequency response of the electrochemical system. The first impedimetric biosensor based on MIP technology with ultrathin insulating membranes was described by Panasyuk and co-authors in 1999 [177].

Special attention has been given to capacitance/impedance technique due to its outstanding properties in terms of high sensitivity, no need for labelling and possible real time monitoring (Table 3).

Recently, in 2016, Khan reported a bulk imprinting assay for Protein A (PA) detection in point of care. The MIP was developed on single walled carbon nanotubes (CNTs)-based screen printed electrodes [110]. The imprinted stage consisted in the mixture of the monomer (3-aminophenol) with PA following its electropolymerization by CV technique. The template was removed from polymeric matrix by proteolytic action of an enzyme. The control of the surface modification and PA detection was followed by EIS. Furthermore, more research work should be performed in order to get a reliable biosensing device with suitable thickness, homogeneous and stable polymeric surfaces.

In another recent work, Moreira and co-authors presented a MIP sensor synthesized on top of a silver SPE fabricated by PCB technology to detect a protein, carcinoembryonic antigen, in point-of-care. The protein was entrapped in the polypyrrole polymeric matrix and removed by enzymatic action of proteinase K. The analytical performance of the biomimetic sensor was evaluated by electrochemical techniques. The sensor shows linear response after 0.05 pg/mL against logarithm concentration [101].

EIS is considered a powerful tool to investigate dynamics of the bound or charge transfer in bulk or at interfacial region of such system. The non-destructive measurements make EIS a promising tool for studying MIP biosensing devices without perturbing its operation.

## 5. Labelling Methods

Commonly, electrochemical detection approaches have fallen into two main categories: labeled and label-free (direct) detection systems. According to the definition, the former rely on the attachment of a label to the molecule of interest to be detected either by its molecular presence or activity. This chemical (covalent bonding) or temporary intermolecular interaction can potentially promote changes on their intrinsic properties and, thus, produce an electrical signal. Instead, a label-free detection system is defined as an approach that do not require the use of biological or chemical receptors (colorimetric, fluorescent, luminescent or radiometric), in order to afford measurements [183,184,185,186].

### 5.1. Label-Free MIP-Based Sensors

Recent trends in the development of sensor structures focus on the use of inexpensive plastic materials, including sensor fabrication on long and continuous sheets of plastic film and in their incorporation into single-use disposable devices. As label-free detection instrumentation becomes more simple and compact, its integration in other biochemical analysis methods, as liquid chromatography, fluorescence assays, and mass spectrometry, will turn the systems capable of characterizing biomolecules with more complex information. The advances in sensors concept, inspire the continuous replacement of label-based assays, like devices based on fluorescence labelling, radiolabels, among others, with label-free detection methods [185]. Unlike what happens with label-based technologies, that simply allow to confirm the presence or absence of a detector molecule, the main advantage of label-free detection is to provide more detailed and direct information (such as selectivity, affinity, stoichiometry, kinetics or, thermodynamics of an interaction), since these methods only investigate native proteins and ligands that constitute a sample [176,185,186,187]. Furthermore, a major advantage of label-free detections is their high sensitivity enabling real-time detection and, thus, simplifying the time and effort of assay development [186,188].

Label-free biosensors have promoted a great advance in different fields, as in material sciences, computational design and nanofabrication. Due to physical properties of some target analytes in terms of dimension, electrical impedance or dielectric permittivity their determination is perhaps difficult to achieve, making it still necessary to rely on label detection.

### 5.2. Labeling MIP-Based Sensors

Several nanomaterials can be used as a label in order to improve the sensitivity of MIP-based sensors. These materials include AuNPs, quantum dots (QDs), CNTs, nanowires, magnetic nanoparticles, graphene, among others. Some research work reports the effect of the presence of CNTs and graphene in the overall performance of MIP sensors.

Bai et al. [189] described an ultrasensitive electrochemical sensor for determination of diethyl-stilbestrol. They conjugated the AuNPs and MWCNT-chitosan composite by drop casting on GC electrode in order to increase the surface area promoting an enhancement of the electron transfer rate, and finally amplifying the sensor signal. In addition, sol-gel MIP was then electrodeposited GC electrode surface. The sensor was characterized using CV and EIS. Electrochemical measurements were performed via DPV. Under optimal conditions, the device showed a detection range of 1.0 × 10^−10^–1.0 × 10^−6^ mg/mL with a LOD of 24.3 fg/mL [189]. Wang and co-authors used a similar strategy, AuNPs and carboxylated MWCNT dropcasted on GC electrode, to produce a MIP sensor for olanquindox (OLA). The MIP was obtained by electropolymerization using OLA as a template and o-PD as monomer, as the novel sensor showed an LOD of 2.7 nmol/L [190].

A biomimetic sensor for sunset yellow detection was created with β-cyclodextrin as a monomer and functionalized with AuNPs and magnetic graphene oxide. The analytical performance of the hybrid nanomaterials was evaluated by CV and EIS. The linear range of the biomimetic sensor after optimization was from 5.0 × 10^−9^ to 2.0 × 10^−6^ mol/L and the LOD of 2.0 × 10^−9^ mol/L [191].

Overall, we can conclude that these nanomaterials show good biocompatibility, special catalytic activity and the convenience of a controlled fabrication process. They can improve the performance of the sensor, such as sensitivity, selectivity and so on.

## 6. Conclusions and Perspectives

This review describes different strategies to produce MIP materials that are combined with electrochemical transduction to fabricate advanced biomimetic sensors. These assays show several advantages including: (i) simple design and procedures; (ii) low price; (iii) portability; (iv) miniaturization and (v) point-of-care.

In general, this paper reviews several technical novelties to construct MIP electrochemical biomimetic sensors for screening different target compounds. In particular, electrochemical sensors show several advantages that have been addressed herein in detail. These devices provide the possibility of multi-analyte measurements, with a low response time, being a promising approach for screening different target analytes as proteins, antibiotics, pesticides, among many others. In addition, these sensors enable multi-target detection with minimal labour and small sample volumes.

One of the major challenges in MIP technology is the ongoing enhancement of MIP selectivity/sensitivity for biological targets. Appropriate chemical conditions and polymer materials compatible with physiological requirements can preserve the natural structure and function (e.g., protein conformation, enzyme activity) of the analyte. MIP synthesis has proven its maturity but continuous research efforts remain, combining new nanomaterials and nanocomposites for preparing MIPs. This approach relies on the enormous advantages offered by nano-based imprinted materials (e.g., better assembly ability, fine-tunability, favorable mass transfer, better binding capacity and kinetics, superior removal of template). There is an enormous development in this field by using nanostructured MIPs (e.g., nanobeads, nanorods, nanowires) with simultaneous advances in porous materials, like hollow microspheres, which combined can provide competitive and versatile well-designed MIPs to empower the electrochemical response in terms of enhanced performance.

Overall, the sensitivity and LOD can be improved by incorporating conductive nanomaterials and electroactive complexes. Some nanomaterials as AuNPs, CNTs, graphene, nanowires and magnetic nanoparticles have been included in the polymeric matrix of MIP-based sensors providing a synergetic effect between catalytic activity, conductivity and biocompatibility, and improving the transduction signal. Increasingly, the integration of different fields as bioinformatics, nanotechnology, materials science, and synthetic biology will favor the achievement of reliable features in terms of analytical performance of the biomimetic sensors.

Moreover, it is possible to configure the MIP-based sensing devices towards multi-sensing array platforms for simultaneous detection of different target analytes. The results should be compared with standard clinical assays/methodologies.

## Figures and Tables

**Table 1:** Voltammetric transduction for MIP based electrochemical sensors (2014–2016).

Analyte Category	Template/Analyte	Monomer	Electrode	Detection Technique	LOD (M)	Linear Range (M)	Reference
Drugs	Ractopamine	Aminothiophenol	Screen printed electrode	DPV	4.23 × 10^−11^	5.0 × 10^−11^–1.0 × 10^−9^	[137], 2016
	Famciclovir	Methacrylic acid and vinyl pyridine	Carbon paste electrode	CV	7.5 × 10^−7^	2.5 × 10^−6^–1.0 × 10^−3^	[138], 2015
	Artemisinin	Acrylamide	Glassy carbon electrode	CV	2.0 × 10^−9^	1.0 × 10^−8^–4.0 × 10^−5^	[139], 2015
	Dopamine	Aminophenol	Metallic microrod	CV	7.63 ×10^−14^	2.0 × 10^−13^–2.0 × 10^−8^	[140], 2016
	Metronidazole	1,2-dimethylimidazole, dimetridazole, o-phenylenediamine	Nanoporous gold leaf	CV	1.8 × 10^−11^	5.0 × 10^−11^–1.0 × 10^−9^ and 1.0 × 10^−9^–1.4 × 10^−6^	[141], 2015
	Theophylline	4-amino-5-hydroxy-2,7- naphthalenedisulfonic acid	Glassy carbon electrode	CA	0.32 × 10^−6^	0.4–17 × 10^−6^	[142], 2016
	Epinephrine	Pyrrole	Indium tin oxide	DPV	-	1–10 × 10^−6^ and 10–800 × 10^−6^	[143], 2016
	Carbofuran	Methyl acrylic acid	Glassy carbon electrode	DPV	2.0 × 10^−8^	5.0 × 10^−8^–2.0 × 10^−5^	[135], 2015
	Eugenol	Aminobenzenethiol-co-p-aminobenzoic acid	Glass carbon electrode	LSV	1.0 × 10^−7^	5.0 × 10^−7^–2.0 × 10^−5^	[144], 2016
Organic molecules	Cholesterol	Aminothiophenol	Glassy carbon electrode	DPV	3.3 × 10^−14^	1.0 × 10^−13^–1.0 × 10^−9^	[145], 2015
	Melamine	Methacrylic acid	Diazonium-modified gold electrodes	SWV	1.75 × 10^−12^	1.0 × 10^−11^–1.0 × 10^−4^	[146], 2015
	Glyphosate	p-aminothiophenol	Gold electrode	LSV	5 × 10^−15^	5.9 × 10^−15^–5.9 × 10^−9^	[147], 2015
	Ascorbic acid	Polyvinylpyrrolidone	Glass carbon electrode	DPV	3.0 × 10^−6^	10–1000 × 10^−6^	[148], 2015
	Dibutyl phthalate	Methacrylic	Gold electrode	DPV	8.0 × 10^−10^	2.5 × 10^−9^–5.0 × 10^−6^	[134], 2015
Biomacromolecules	Protein A	Aminophenol	SWCNT-Screen printed electrode	SWV	0.6 × 10^−9^	23.8 × 10^−9^–4.76 × 10^−6^	[110], 2016
	Guanine-rich DNA (G-rich DNA)	Methacrylic acid and guanine	MWCNT electrode	DPV	7.52 × 10^−9^	0.05–1× 10^−6^ and 5–30 × 10^−6^	[149], 2016
	Benzo[a]pyrene	Vinylferrocene	Carbon paste electrode	SWV	0.09 × 10^−6^	0.08 × 10^−6^–3.97 × 10^−6^	[133], 2014
	Carcinoembryonic antigen	Pyrrole	Silver-Screen printed electrode	SWV and CV	2.8 × 10^−16^	2.8 × 10^−16^–6.9 × 10^−15^	[101], 2016
	Human serum albumin	bis(2,2′-bithien-5-yl)methan	Gold electrode	DPV	0.25 × 10^−12^	12 × 10^−12^–300 × 10^−12^	[150], 2015
	DNA	Pyridine	Carbon paste electrodes	SWV	1.38 × 10^−6^	0–7.9 × 10^−6^	[151], 2015
	Troponin T	Pyrrole	Screen printed electrode	SWV	1.64 × 10^−13^	2.74 × 10^−13^–2.74 × 10^−12^	[152], 2016

**Table 2:** Potentiometric transduction for MIP based electrochemical sensors (2013–2016).

Analyte Category	Template/Analyte	Monomer	Electrode	Detection Technique	LOD (M)	Linear Range (M)	Reference
Drugs	Azithromycin	Acrylic acid and 2-vinyl pyridine	Graphite electrode	Potentiometry	1.0 × 10^−7^	1.0 × 10^−1^–1.0 × 10^−6^	[157], 2015
	Losartan	Methacrylic acid	Graphene/carbon paste electrode	Potentiometry	1.82 × 10^−9^	3.0 × 10^−9^–1.0 × 10^−2^	[158], 2015
	Clenbuterol	Chitosan	Carbon paste electrode	Potentiometry	0.91 × 10^−11^	1.0 × 10^−7^–1.0 × 10^−12^	[159], 2016
	Taurine	3,4-Ethylenedioxythiophene	Glassy carbon disc electrodes	Potentiometry	-	1.0 × 10^−2^–1.0 × 10^−4^	[160], 2016
	Histamine	Methacrylic acid	Solid phase extraction	Potentiometry	1.12 × 10^−6^	1.0 ×1 0^−6^–1.0 × 10^−2^	[161], 2014
	Carnitine	Vinylbenzyl trimethylammonium chloride and 4-styrenesulfonic acid	Graphite and ITO/FTO	Potentiometry	3.6 × 10^−5^	1.0 × 10^−6^–1.7 × 10^−3^	[162], 2014
	Neopterin	Bis-bithiophene, bithiophene derivatized with boronic acid	Pt disk-working electrode	Potentiometry	22 × 10^−6^	0.15 × 10^−3^–2.5 × 10^−3^	[163], 2016
	Dopamine	Acrylamide grafted MWCNTs	Cu electrode surface	Potentiometry	1.0 × 10^−9^	1.0 × 10^−9^–1.0 × 10^−5^	[164], 2014
	Urea	Poly(methyl methacrylate)	ISFET	Potentiometry	1.0 × 10^−4^	1.0 × 10^−4^–1.0 × 10^−1^	[165], 2016
	Memantine hydrochloride	Methacrylic acid	—	Potentiometry	6.0 × 10^−6^	1.0 × 10^−5^–1.0 × 10^−1^	[155], 2013
	Chlorogenic acid	Pyrrole	Graphite electrode	Potentiometry	1.0 × 10^−6^	1.0 × 10^−2^–1 × 10^−6^	[166], 2016
Biomacromolecules	Prostate specific antigen	Vinylbenzyl(trimethylammonium chloride), vinyl benzoate	Conductive carbon over a syringe	Potentiometry	5.8 × 10^−11^	5.83 × 10^−11^–2.62 × 10^−9^	[167], 2016
	Carcinoembryonic antigen	11-Mercapto-1-undecanol	Gold electrode	Potentiometry	2.8 × 10^−12^	2.8–8.3 × 10^−11^	[168], 2016

**Table 3:** Impedimetric/Capacitive transduction for MIP based electrochemical sensors (2013–2016).

Analyte Category	Template/Analyte	Monomer	Electrode	Detection Technique	LOD (M)	Linear Range (M)	Reference
Drug	Carnosine	Carboxy and 18-crown-6 ether and bis(2,20-bithien-5 yl)methane	Gold electrode	EIS	20 × 10^−6^	0.1 × 10^−3^–0.75 × 10^−3^	[178], 2016
	Theophylline	Pyrrole	Silicon substrates	EIS	-	0.1 × 10^−9^–1.0 × 10^−6^	[179], 2015
	Aflatoxin B1	Ovalbumin and glutaraldheide	Gold electrode	EIS	6.3 × 10^−12^	3.2 × 10^−6^–3.2 × 10^−9^	[180], 2016
Biomacromolecule	Prostate specific antigen	Dopamine	Gold electrode	EIS	2.94 × 10^−14^	2.94 × 10^−9^–2.94 × 10^−12^	[181], 2016
	Protein A	Aminophenol	SWCNT-screen printed electrode	EIS	16.8 × 10^−9^	23.8 × 10^−9^–2.38 × 10^−6^	[110], 2016
	Carcinoembryonic antigen	Pyrrole	Silver- screen printed electrode	EIS	2.8 × 10^−16^	2.8 × 10^−16^–6.9 × 10^−15^	[101], 2016
	Carnitine	3,4-ethylenedioxythiophene (EDOT)	Carbon-cellulose paper	EIS	2.15 × 10^−10^	1.0 × 10^−8^–1.0 × 10^−3^	[182], 2016
